# Hyperspectral microarray scanning: impact on the accuracy and reliability of gene expression data

**DOI:** 10.1186/1471-2164-6-72

**Published:** 2005-05-11

**Authors:** Jerilyn A Timlin, David M Haaland, Michael B Sinclair, Anthony D Aragon, M Juanita Martinez, Margaret Werner-Washburne

**Affiliations:** 1 Sandia National Laboratories*, P.O. Box 5800, Albuquerque, NM, 87185, USA; 2 Lovelace Respiratory Research Institute, 2435 Ridgecrest SE, Albuquerque, NM, 87108, USA

## Abstract

**Background:**

Commercial microarray scanners and software cannot distinguish between spectrally overlapping emission sources, and hence cannot accurately identify or correct for emissions not originating from the labeled cDNA. We employed our hyperspectral microarray scanner coupled with multivariate data analysis algorithms that independently identify and quantitate emissions from all sources to investigate three artifacts that reduce the accuracy and reliability of microarray data: skew toward the green channel, dye separation, and variable background emissions.

**Results:**

Here we demonstrate that several common microarray artifacts resulted from the presence of emission sources other than the labeled cDNA that can dramatically alter the accuracy and reliability of the array data. The microarrays utilized in this study were representative of a wide cross-section of the microarrays currently employed in genomic research. These findings reinforce the need for careful attention to detail to recognize and subsequently eliminate or quantify the presence of extraneous emissions in microarray images.

**Conclusion:**

Hyperspectral scanning together with multivariate analysis offers a unique and detailed understanding of the sources of microarray emissions after hybridization. This opportunity to simultaneously identify and quantitate contaminant and background emissions in microarrays markedly improves the reliability and accuracy of the data and permits a level of quality control of microarray emissions previously unachievable. Using these tools, we can not only quantify the extent and contribution of extraneous emission sources to the signal, but also determine the consequences of failing to account for them and gain the insight necessary to adjust preparation protocols to prevent such problems from occurring.

## Background

Since their introduction in 1995 [1], DNA-based microarrays (also known as genechips) have driven an explosion in functional genomic analyses. All varieties of microarrays have in common the ability to perform binary comparisons of gene expression for a large number of genes simultaneously in a microchip format [2-6].

In theory, biological changes should define the limitations of microarray technology, but unfortunately, technological issues have frequently limited the usefulness of microarray data. Non-biological factors including printing artifacts, dye-gene interactions, background emissions, and slide-to-slide variations significantly reduce the ability to accurately monitor changes in gene expression in microarray experiments [4,7-10]. These experimental factors are common and often laboratory dependant due to the complicated multi-step procedures used in the production, hybridization, and analysis of microarrays. In attempts to minimize the effect of variability due to non-biological sources, a variety of statistical analyses [11,12], normalization techniques [13-15], and metrics for image quality [16] have been proposed. However, all these analysis techniques have two assumptions: 1) that the only contributors to the signal within a printed spot are the labeled DNA and a uniform background due to emission of the glass substrate and 2) that background fluorescence surrounding the spot is the same as background fluorescence under the spot, despite evidence that this assumption may not be valid [7,12,17].

Neither of these assumptions can be validated with current commercial microarray scanners. Commercial scanners are univariate instruments; that is they use filters to pass all photons emitted in a specific wavelength range to a single point detector. This mode of operation can be fast, but it does not allow discrimination of photons by emission source. Thus, it is not possible to distinguish two photons of the similar wavelength that arise from different emitting species if they are passed through the filter for that channel. Many problems that plague microarrays (inaccuracies in background correction, dye-gene effects, skew toward one channel, dye crosstalk, and contaminating fluorescence) cannot be accurately assessed in data from filter-based microarray scanners due to this limitation and this can lead to erroneous data [9,17].

To address these issues, we have developed a hyperspectral-imaging microarray scanner [18] that allows the simultaneous quantification of all fluorescent species, including the spot-localized background leading to a significant improvement in the accuracy of microarray data. The hyperspectral scanner (HSS) coupled with multivariate data analysis provides in-depth understanding of the signal detected by traditional microarray scanners and can promote improvement in microarray technology and actually improve the quality of microarray data. The benefits of an additional dimension of spectral information for material science, cytogenetic, and histological applications [19] and live-cell microscopy [20] have been reviewed. However, until our report, hyperspectral imagers have not demonstrated the sensitivity or speed of commercial microarray scanners or the multivariate data analysis capabilities necessary to extract sufficient information from the complex data [21-23]. This paper presents the use of the HSS and multivariate data analysis to understand three anomalies commonly seen in microarray data: skew toward the green channel, dye separation, and high, variable background signal. The unique capability of HSS technology to identify and correct for the presence of these phenomena improves the reliability and accuracy of gene expression data.

## Results and Discussion

### Hyperspectral imaging and multivariate data analysis

**Figure 1:**
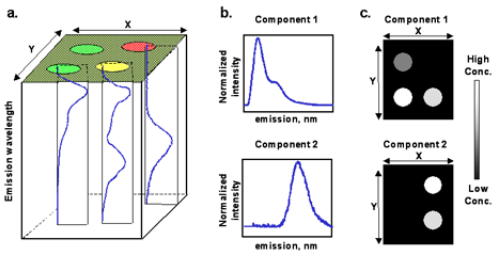
**Illustration of hyperspectral data cube and multivariate analysis results**. **(a) **Three-dimensional hyperspectral data cube for an idealized two-component microarray containing emission only from labeled DNA within the printed spots. Each pixel in the x-y image plane contains an entire fluorescence emission spectrum from 550–900 nm. **(b-c) **Results of multivariate curve resolution on two-component sample hyperspectral data cube shown in A. **(b) **Pure component spectra identify "what" species are fluorescing in an image. **(c) **Corresponding component concentration maps show "where" and "how much" of each component in Fig. 1b is present.

The HSS we have developed is optimized for imaging printed DNA microarrays and excites a sample with a single laser, typically at 532 nm, while recording the emission over a wavelength range from 550–900 nm in approximately 0.75 nm increments to create a hyperspectral data cube (Fig. 1a). Details of the optical design and characterization of this line-imaging system have been published elsewhere [18]. Additional lasers are available and the wavelength range and spatial and spectral resolution are adjustable. The sensitivity and dynamic range of this HSS is the same as or better than the commercial microarray scanners we have tested for dyes emitting in the green channel of commercial systems such as Cy3 [18]. Typically, red emitting dyes like Cy5 are not optimally excited by our 532 nm laser, but based on its excitation spectrum, we are achieving ~5–8% excitation of Cy5. In comparison studies between an Axon 4000B microarray scanner exciting Cy5 with 633 nm light and the HSS exciting Cy5 with 532 nm light (data not shown) the HSS was found to be a factor of 6 less sensitive for Cy5 than commercial microarray scanner. However, the signal acquired from Cy5 is sufficient for quantitation by the multivariate algorithms. In addition, for the studies reported in this publication the focus is predominantly on the green channel emissions. In its current configuration the HSS scanner operates at a slightly slower speed of data acquisition (scanning at a maximum rate of 0.07 mm/s for 10 μm resolution) but this disadvantage is compensated for by the more accurate quantification and increased specificity of the fluorescent signal, especially in the presence of contaminants. The CCD readout rate is the limiting factor in the current HSS and newly available charge-coupled device (CCD) detector electronics could allow the HSS to scan at speeds up to twice the speed of commercial scanners.

**Figure 2:**
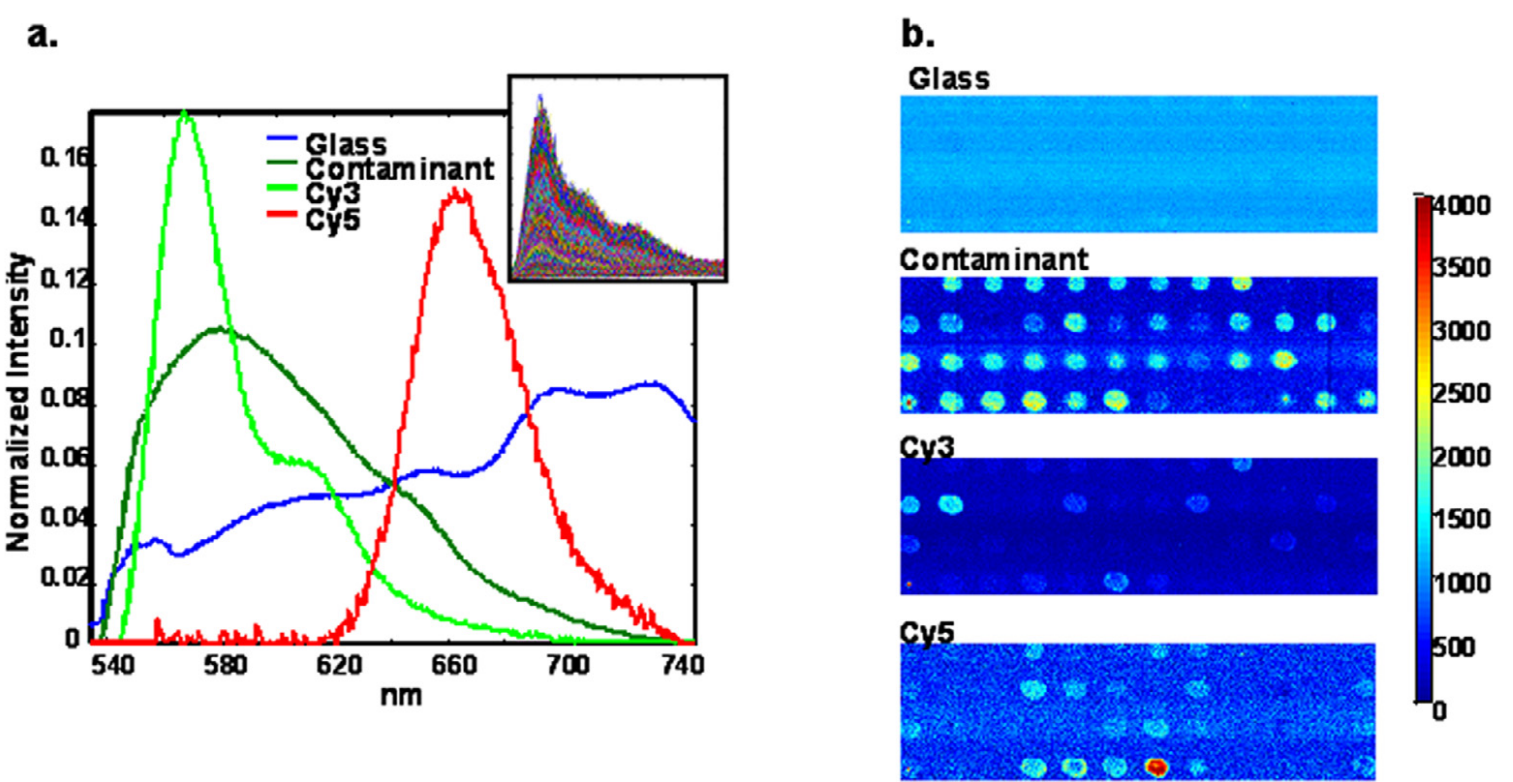
**MCR analysis results of cDNA microarray exhibiting contamination fluorescence**. Fluorescence of all species was excited simultaneously in a single scan with 532 nm laser. **(a) **MCR extracted pure component spectra representing glass, Cy3, Cy5 and a contaminant. Inset shows ~30000 raw spectra in the image data cube used to determine the pure component spectra. **(b) **Extracted concentration maps corresponding to each of the pure component species in Fig. 2a. Resulting HSS intensities have been scaled to match the individual channel intensities seen on the Axon 4000B microarray scanner.

A typical hyperspectral data cube contains tens of thousands to millions of spectra and spectral and spatial relationships cannot be readily visualized (Fig. 1a). To reduce the data to a simpler representation of the important features, HSS data are analyzed using multivariate curve resolution (MCR), a powerful factor analysis technique based on a constrained alternating least squares procedure [24]. MCR algorithms, when applied to fluorescence emission images, use correlations between variables to extract 1) representative pure-component spectra of the all the emitting species (Fig. 1b) and 2) independent concentration maps depicting the spatial location of these species and their relative concentrations from the highly overlapped spectral data (Fig. 1c). The MCR analysis assumes that the number of non-noise components is known or can be estimated and requires initial estimates to either the spectra or concentrations.

Principal component analysis, a closely related and popular multivariate analysis technique for biological data [25], typically provides an accurate determination of the number of non-noise components, but often cannot provide easily interpretable pure component spectra underlying complex spectral image data due to rotational ambiguity. For these studies involving microarray data, principal component analysis was used to determine the number of non-noise components and to provide initial estimates of the spectral shapes for the MCR algorithm to iterate upon. The application of non-negativity and equality spectral constraints within the MCR algorithms facilitates physically meaningful solution of spectral components. The benefits of MCR over more traditional univariate (band integration, band ratios) and other multivariate (least squares, linear unmixing) analysis methods include the ability to separate overlapping spectral emissions, discover the pure-component spectra with little or no information given *a priori*, and model unknown spectral contributions such as interferrents, backgrounds, and instrument artifacts. Applications of MCR for vibrational spectroscopic data have been outlined in a recent review [26]. Recently, members of our group have developed efficient, automated multivariate statistical analysis algorithms to analyze large X-ray hyperspectral image data using desktop computers [27]. We have optimized these algorithms for fluorescence image data from the HSS [28,29].

Throughout this paper, emissions from within the area of the printed DNA spots are referred to as spot-specific emissions and emissions that are not spot-specific are referred to as background emissions. Also, ratio images generated by commercial microarray scanners are referred to as R/G (Red/Green) images and images generated by HSS are referred to as Cy5/Cy3 images, because the commercial scanner image is constructed from the intensities in the red and green channels regardless of source, whereas the HSS and data analysis produces true images of the Cy5 and Cy3-labeled cDNA that are uncontaminated by emission from other species.

### Spot-localized emissions: Skew to toward the green channel

Earlier work has shown the presence of contaminant fluorescence in the green channel of many common types of microarray slides [17]. The HSS was used to confirm the presence of the green contaminant in slides from a variety of laboratories (four different commercial sources and in-house printed arrays from at least four independent laboratories) after hybridization. This contaminant is introduced to the slide during printing and can be recognized in the raw images as a variable, weak green channel signal that may be observed in all spots. In our investigations we examined slides from at least three laboratories printed using DMSO printing methods and did not identify a green contaminant in any of the spots on these slides.

G_total _= G_Cy3 _+ G_glass _+ G_cont _   Eq. 1

Commercial filter-based microarray scanners will confound signal from a spot-localized green contaminant with signal from the labeled cDNA and glass substrate in the green channel. This is because the green signal intensity recorded for a pixel with a spot-localized green contaminant is the sum of all the green fluorescence intensities, including the glass emission (a relatively constant small value), the contaminant (a variable value), and the labeled hybridized cDNA. This extra green intensity has no affect in the red channel and little effect on spots with high green channel signal, but contributes significantly to the signal from spots with weak and medium green-channel intensities. As illustrated in Equation 1 data obtained from slides with spot-localized contaminating fluorescence will report green channel intensities that are falsely high (G_total _is the total signal acquired in the green channel; G_Cy3_, G_glass_, and G_cont _are the signals arising from the Cy3 labeled DNA, glass substrate and contaminant, respectively.) Unfortunately the effect of spot-localized contaminant emission cannot be corrected in commercial scanner microarray data using standard background correction procedures that estimate background signal for a spot from the signal around the spot and thus it leads to errors in the calculated expression ratios (R/G). The detrimental effect of the contaminant decreases with increasing green-channel intensities but can easily account for skews toward the green channel, dye-gene effects, unsuccessful dye-flip experiments, and highly variable low intensity data observed in many microarray experiments.

**Figure 3:**
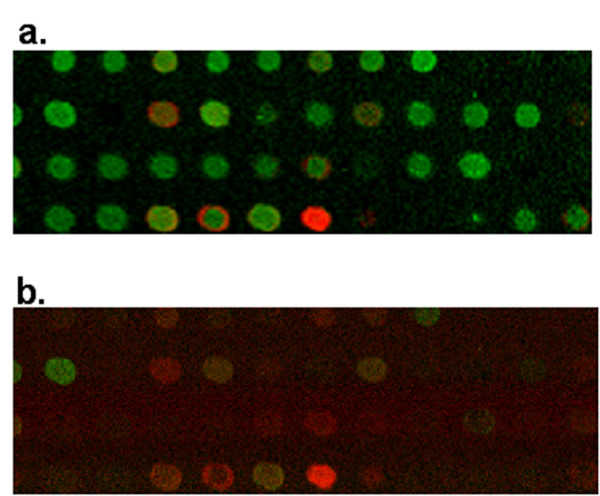
**Green contaminant presence alters resulting image from commercial scanner**. Comparison of resulting images from two scanners with green contaminant present. **(a) **Red/Green ratio image generated from Axon 4000B microarray scanner depicting most spots as green indicating the control gene was expressed to larger extent than the treatment. **(b) **Cy5/Cy3 ratio image generated from HSS concentration maps depicting only a few spots as differentially expressed.

Figure 2 shows the results of multivariate data analysis of a hyperspectral image from a microarray slide with contaminating fluorescence. Because the HSS can isolate the contaminant emission spectrum, true pure-component concentration maps can be generated and the resulting images of the Cy3 labeled cDNA are contaminant-free. Using the optical filter functions of the Axon 4000B microarray scanner, HSS images can be scaled to match the total intensity of each of the commercial scanner channels, thus providing a direct comparison to the commercial scanner results. These scaled concentration maps are used to calculate an accurate Cy5/Cy3 image that gives spot intensity values without contributions from the glass and contaminant emissions. The R/G ratio constructed from the commercial scan (Fig. 3a) and the more accurate Cy5/Cy3 ratio image of the same area on the slide (Fig. 3b) show the difference is dramatic, with 75% of the spots having ratios in error of a factor of 2 or more due to the presence of the green channel contaminant. These errors would change the basic conclusion from the data that most genes in the test sample were down-regulated relative to the control when, in fact, the correct conclusion should be that most genes in the test sample were either up regulated or not differentially expressed. The fluorescence contribution of the contaminant cannot be corrected with current commercial technology due to extreme spectral overlap and the variability of the spot-localized contaminant concentration. Image thresholding could be useful if the contaminant contribution were known and fairly constant but even low levels of contaminant fluorescence would require high thresholds be set. For example, a maximum of 100 arbitrary fluorescence units of spot-localized contaminant signal would require a threshold of 500 arbitrary fluorescence units be set in an attempt to maintain errors in the 20% range.

### Spot-localized emissions: Dye separation

"Dye separation" is a phenomenon referred to in the microarray literature as a ring of one color surrounding the other, typically red around green [16]. Although this phenomenon is seen in published images, there is no satisfactory explanation for what causes the labeled cDNA to hybridize with a dye-specific spatial pattern. Other spatial patterns of hybridization, e.g., doughnuts and coffee rings are well documented, but spatial anomalies theoretically should affect both dyes within a spot to the same extent.

**Figure 4:**
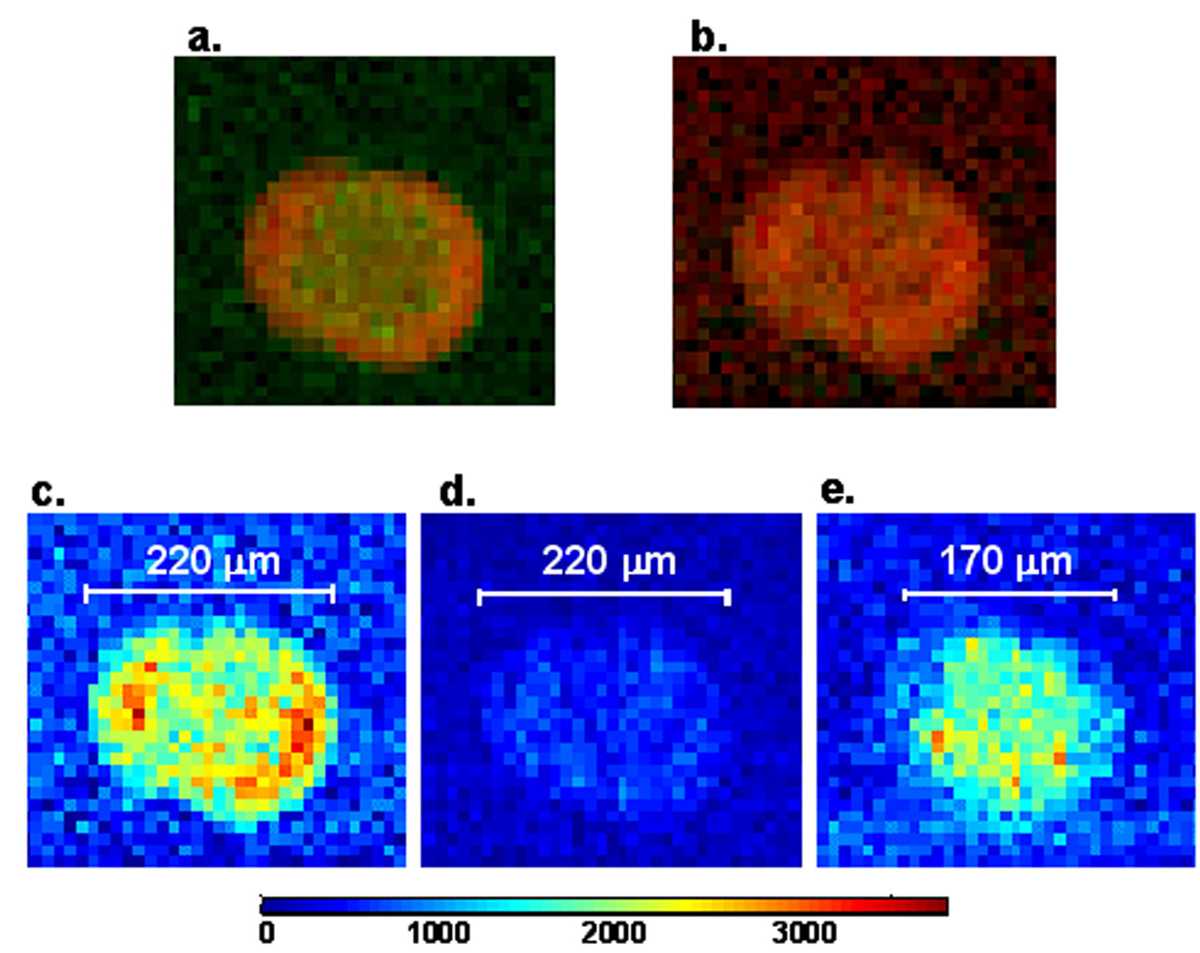
**Images illustrating dye separation**. **(a) **Red/Green ratio image generated from Axon 4000B microarray scanner showing the red ring characteristic of apparent dye separation. **(b) **Cy5/Cy3 ratio image generated from HSS concentration maps showing uniform Cy5 and Cy3 distribution throughout the spot. **(c-e) **Individual component concentration maps resulting from the multivariate analysis of the HSS image data highlighting the difference in spot diameter of the brighter contaminant that results in the apparent dye separation when imaged with a filter-based microarray scanner. All fluorescence emissions in the hyperspectral images were excited simultaneously in a single scan. **(c) **Cy5 concentration map. **(d) **Cy3 concentration map. **(e) **Green contaminant concentration map.

Apparent dye separation was visible in some spots on the microarray slides we examined with the green contaminant. Figure 4a shows a R/G ratio image from a typical spot exhibiting dye separation scanned on an Axon 4000B scanner. From this image, the Cy5-labeled cDNA appears to have hybridized in a circle around the area where the Cy3-labeled cDNA hybridized. HSS analysis revealed that the spot-specific signal was from three sources: Cy5-labeled cDNA, Cy3-labeled cDNA, and green contaminant. The individual concentration maps generated from the multivariate analysis of the HSS image data demonstrate that, for this spot, the diameters of Cy5 and Cy3 hybridization are equal, although little Cy3 is present (Fig. 4c–d). The contaminant emission, which is brighter than the Cy3, is present in a smaller diameter circle (Fig. 4e) and the result of this localization difference is a spot with a bright green center and a red ring on commercial scanners. This size difference most likely occurs because of differences in surface tension of the printed cDNA and contaminant when drying or differences in charge of the cDNA and contaminant. Figure 4b shows an accurate Cy5/Cy3 image of this spot created from the HSS concentration maps. The original Axon data produced a ratio of Cy5/Cy3 medians of 3.0 and ratio of Cy5/Cy3 means of 3.0 while the more accurate HSS data gives rise to ratios of medians and means of 7.7 and 7.5, respectively, for this particular spot.

### Background emissions

In microarray experiments, mean or median intensities are calculated per spot to determine expression ratios. These spot intensity values are typically corrected for background emission using methods that subtract local or global estimates of background contributions. Various background correction methods exist in data analysis software, but all of these methods make one critical assumption about the data – that the background emissions are the same outside the spot as they are under the spot. This assumption is valid in an ideal situation where a perfectly homogeneous glass slide is the sole source of background emissions and the printed DNA spot is sufficiently thin and non-scattering so as not to interfere with the excitation of the glass beneath it. Unfortunately this assumption is rarely valid. Researchers have shown the results of microarray experiments are very dependant on background subtraction methods used and have theorized that local background values are not representative of the true background emission in a spot, leading to erroneous values and negative spot intensities [30]. Options such as not correcting for background, using a global background value for every spot, or using negative control values as a background can be successful in some cases, but are not robust.

Using the HSS, we have explored background emissions on many printed microarrays spanning a variety of preparation protocols. The unique ability to identify and isolate all emission sources and model the background for each pixel directly from the spectra allows us to generate pure-concentration maps of the dyes of interest without contributions from background sources. In every microarray we have scanned (9+ different labs, in-house and commercially printed slides) the background was different under the spot than around the spot. This difference can vary from a subtle decrease in intensity under the printed spot to a much more predominant intensity variation that seriously affects the accuracy of the data. Understanding the background emissions is essential to ensuring that appropriate background correction techniques are used when scans are to be performed with commercial scanners. This increased understanding of background emissions also provides the feedback necessary to alter the preparation process to minimize the background emissions present on microarray slides.

**Figure 5:**
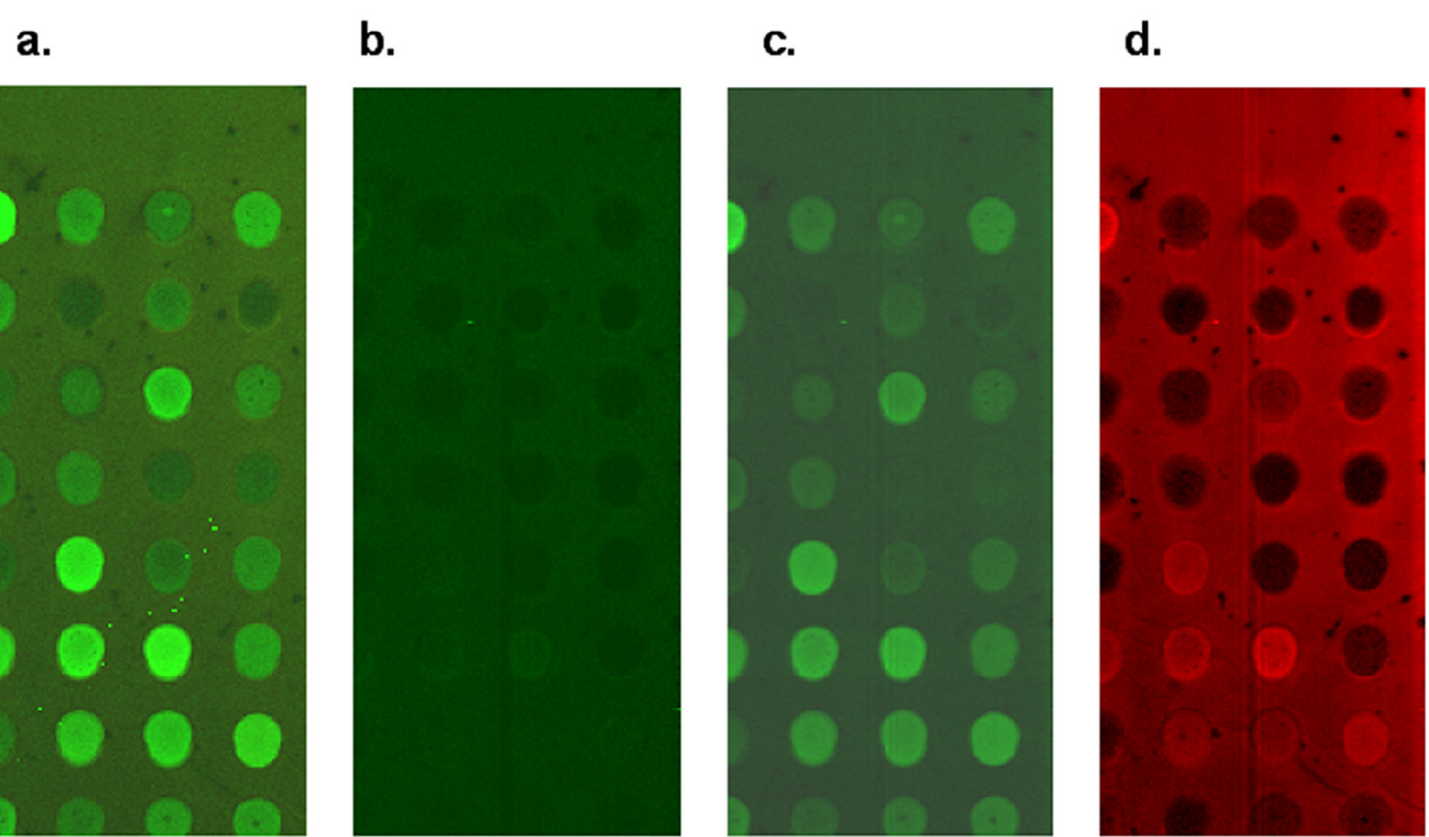
**Images illustrating background problems**. All images are of same area on the microarray. **(a) **Red/Green ratio image generated from Axon 4000B microarray scanner. The green channel intensity dominates background and spot signal. **(b-d) **Individual component concentration maps extracted from multivariate analysis of HSS image data. All fluorescence emissions in the hyperspectral images were excited simultaneously in a single scan. **(b) **Glass concentration map. Note that the spots all appear slightly dimmer than the background. **(c) **Cy3 concentration map. **(d) **Cy5 concentration map.

Figure 5a shows the R/G ratio image of a portion of a microarray with spatially variable, high background emissions and poor spot-specific signal in the red channel. Although only a small area is shown, the entire array contains significant variations in background intensities and patterns. Multivariate analysis of the spectra from a HSS image determines that the emission outside of the spots is predominantly from Cy5-labeled cDNA, but also includes contributions from Cy3-labeled cDNA as well as the glass substrate. In this case the non-specific interaction of the labeled cDNA with the glass substrate is most likely caused by inadequate blocking procedures. Non-specifically bound cDNA is not expected to contribute to the intensities within a spot and therefore local or even global subtraction methods would lead to spot intensities that are too low and even negative. The critical advantage of HSS analysis in this situation is that pure emission spectra are quantified and spot intensities from these images do not need separate background correction.

Two additional points should be noted about Figure 5. First, in the HSS Cy3 concentration map, Cy3 is present both in and outside of the printed cDNA spots. This is in contrast to the Cy5-HSS image, in which Cy5 is absent from many spots. We have observed a slight spectral shift in the Cy5 emission maximum under these conditions compared to other slides with successful Cy5 hybridization, suggesting that the Cy5 outside the spots may contain significant amounts of residual, unincorporated dye. Cy5 and Cy3 both exhibit a spectral shift in emission maxima upon incorporation into cDNA. This effect is illustrated in Additional File 1.

Additional File 1 compares the MCR extracted spectra from a spotted array containing only Cy5-dCTP in the spots and a second spotted array containing only Cy5-cDNA in the spots showing the effect the molecule Cy5 is attached to can have on the emission maxima of Cy5. Second, the HSS image of the glass concentration shows another phenomenon: the glass intensity is lower under very bright spots (spots with intensities > 6000 arbitrary fluorescence units on the commercial scanner we utilized) than outside of the spots. This difference is slight but consistent. We believe this is due to scattering or absorption of the laser light by the printed spot, decreasing the irradiance of the glass beneath the DNA spot.

We also observed several microarrays with high background in smears across the slide. (data not shown) In those cases, the spectra from the smears did not resemble Cy3 or Cy5, but, instead, were from a green contaminant. The smears persist across the surface of the spot and contribute to additional signal intensity in the Cy3 channel. No background correction method can adequately correct for this emission and unless the contribution from this contaminant is modelled and removed from the data, the analyzed microarray data will not be reliable.

## Conclusion

The microarrays utilized in this study consisted of commercial preprinted slides or slides printed in-house using widely accepted techniques, and thus are representative of a wide cross-section of the microarrays currently employed in genomic research. The results presented in this paper demonstrate that three common anomalies cited in the microarray literature can be explained by extraneous emission sources, reinforcing the urgent need for careful attention to detail to recognize the presence of extraneous emissions that reduce accuracy of existing microarray data.

Our work illustrates the impact hyperspectral scanning can have on the accuracy and reliability of genomic data. Hyperspectral imaging, together with multivariate data analysis, is a critical tool for understanding and characterizing the sources of microarray emissions after hybridization. Our state-of-the-art HSS and multivariate algorithms permit the simultaneous identification and quantification of contaminant and background emissions in microarrays, thus markedly improving the reliability and accuracy of these data. Using these tools, we can obtain accurate concentration maps that are corrected for the effect of contaminant emissions. Perhaps more importantly, this approach provides the capability to quantify the extent and contribution of extraneous emission sources to the signal, the consequences of failing to account for them, and the insight necessary to adjust preparation protocols to prevent such problems from occurring. The utility of the HSS technology coupled with multivariate data analysis is not limited to microarrays, but could improve the understanding of data from a variety of applications based of fluorescence imaging and microscopy. In addition to providing more accurate measurements, the HSS also has potential to increase the throughput and improve analysis of microarray experiments by providing quantitation of multiple overlapping dyes. Future work will demonstrate this capability with microarrays and multiple fluorescence imaging applications.

## Methods

### Microarray slide preparation

The data presented represent timepoints from a large experiment performed to study gene expression in yeast cells exiting the stationary phase. Wild-type *MATα*S288c cells (ATCC) were grown in 100 ml YPD+A at 30°C for 7 days (stationary phase) with aeration. Cells of a 7-day culture were resuspended in fresh media prewarmed to 30°C and samples were taken at various timepoints. 7-Day cells sampled before refeeding served as the reference timepoint 0. Approximately 4 × 10^9 ^cells were collected to yield 50–200 μg total RNA. The RNA protocol is described in the web supplement. . Twenty μg of RNA was used for each labeling reaction. Cy3 and Cy5 (Amersham) labels were incorporated into reference and experimental cDNA (green and red, respectively) via first strand cDNA synthesis.

For the slides shown in this paper commercially printed CMT S288C yeast v. 1.32 arrays (Corning) were used although additional slides from other laboratories were investigated and are consistent with our findings for these arrays. The slides were pre-hybridized, hybridized, and post-processed according to procedures described in previously published methods [17].

### Microarray scanning and analysis using commercial microarray scanner

Hybridized glass microarray slides were scanned with 10 μm spatial resolution using an Axon 4000B microarray scanner. Instrument settings were as follows: 100% laser power and PMT settings of 600–650 for both the red and green channels. Resulting images were analyzed using GenePix 3.0 or 4.0 (Axon) to locate and quantify the printed spots. Grids used to define spot diameters were aligned manually and diameters were adjusted to match actual spot diameters (180–240 μm). Care was taken to flag spots that were visibly compromised (dust, scratches, smears, etc.) as "bad' and these spots were not used in calculating statistics or in any future analyses. Background levels were calculated locally for each spot using the median of the background pixels. The spot intensities reported are the median values after local background subtraction.

### Microarray scanning using hyperspectral microarray scanner

Following the scans with the commercial scanner, areas on the same slides were scanned using the HSS [18]. All fluorescence was excited with a 532 nm laser (<20 mW total power dispersed over a 1 mm × 0.010 mm area for line imaging) and the emission spectrum from 550 to 900 nm was collected in line scanning (push-broom) mode with a scan speed of 0.04 mm/s and a CCD rate of 4 frames/sec with EMCCD gain set to 128. This scanning protocol produced images with 10 μm spatial resolution in the motion direction. A bin by 2 configuration was used in the vertical dimension of the CCD in order to assure a spatial resolution of 10 μm in that dimension using a 10× objective (Nikon PlanApochromat) and taken into consideration our overall system magnification (6× with 10× objective). Instrument control/data collection was accomplished using custom software written in the C++ environment.

All image preprocessing and multivariate analysis was performed using in-house written Matlab software. (Matlab v 6.5, Mathworks, Inc) HSS images were preprocessed to locate and remove cosmic events using a modified, 5-point median filter over the immediate area of a cosmic spike only. To correct for spectral image curvature and to calibrate the wavelength axis reference images of neon and krypton gas discharge lamps were used. Signals from both lamps were combined for accurate calibration over the large spectral range. Principal component analysis was performed on the resulting preprocessed images and the number of spectral components was determined by inspecting the eigenvalues in a Scree plot [31]. A constrained, alternating least squares MCR analysis was performed using the number of components from the PCA analysis plus an additional offset (linear baseline) component. The MCR algorithm also requires initial estimates for the spectra and/or the concentrations. The algorithm was initialized with either the positive values of the principal components or spectra identified from previous image analyses. Non-negativity constraints were applied to all components for both concentrations and spectra and spectral equality constraints were applied to the offset component throughout the analysis and to portions of other component spectra as needed to prevent physically unrealistic solutions (i.e. Cy5 emission signal at wavelengths <590 nm or spectral or concentration nonnegativity). All constraints were applied using rigorous least squares methods. One hundred to five hundred iterations were performed in the MCR analysis, although the solution often converged in fewer iterations. Resulting component concentration maps were scaled to be directly comparable to the commercial scanner results by convolving the hyperspectral data recreated by the concentration maps of the emissions of interest with the optical filter function of each of the commercial scanner channels. The results were integrated and scaled to achieve total integrated intensities equal to the per channel values obtained with the commercial scanner. These scaled concentration maps were then written as individual tif files and read into the GenePix software where spots were identified and quantitated for each of the components using the methods presented above for commercial microarray data.

## Authors' contributions

JAT performed the hyperspectral scanning, multivariate data analysis, spectral interpretation, and prepared the manuscript. MBS designed and constructed the hyperspectral microarray scanning and wrote all data acquisition software. DMH conceived the study, developed the multivariate data analysis tools and techniques and aided in the interpretation of the results. ADA performed commercial microarray experiments. MJM performed commercial microarray experiments and data analysis, and assisted in drafting the manuscript. MWW conceived the study, coordinated microarray experiments, provided biological expertise and interpretation, and assisted in manuscript writing. All authors read and approved the final manuscript.

## List of Abbreviations

hyperspectral scanner (HSS)

multivariate curve resolution (MCR)

charge-coupled device (CCD)

electron multiplier CCD (EMCCD)

Red/Green ratio (R/G)

## Supplementary Material

Additional File 1 **Spectral shifts Cy5 upon incorporation into cDNA **MCR extracted spectral profiles of Cy5-dCTP (blue trace) and Cy5-cDNA (green trace). Spectra profiles were obtained from MCR analysis of hyperspectral images from two individual spotted arrays manufactured in-house containing spots of only Cy5 bound to dCTP and Cy5 incorporated into cDNA. Spectral traces are normalized for maximum intensity equal to one Click here for file
