# Supplementary figures and images for: Hyperspectral microarray scanning: impact on the accuracy and reliability of gene expression data
Source: BMC Genomics. 2005 May 11;6:72. doi: 10.1186/1471-2164-6-72 (PMC1156888; doi:10.1186/1471-2164-6-72)

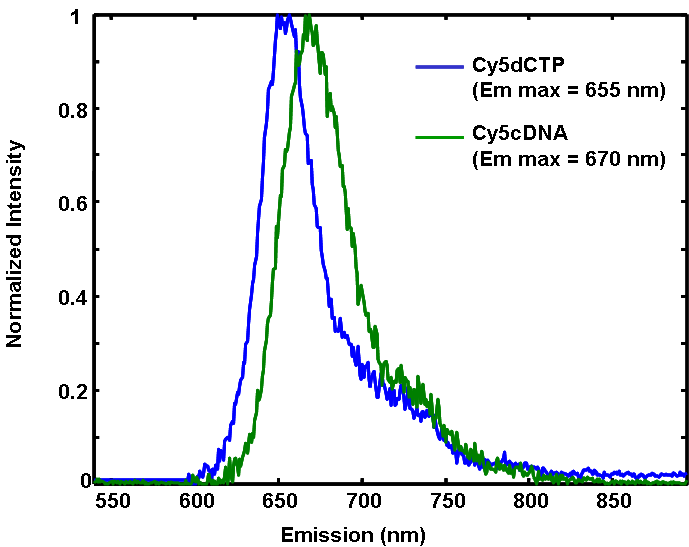

Supplement: Additional File 1 — Spectral shifts Cy5 upon incorporation into cDNA MCR extracted spectral profiles of Cy5-dCTP (blue trace) and Cy5-cDNA (green trace). Spectra profiles were obtained from MCR analysis of hyperspectral images from two individual spotted arrays manufactured in-house containing spots of only Cy5 bound to dCTP and Cy5 incorporated into cDNA. Spectral traces are normalized for maximum intensity equal to one [file 1471-2164-6-72-S1.tiff]
